# Human Schwann Cell Transplantation for Spinal Cord Injury: Prospects and Challenges in Translational Medicine

**DOI:** 10.3389/fncel.2021.690894

**Published:** 2021-06-18

**Authors:** Paula V. Monje, Lingxiao Deng, Xiao-Ming Xu

**Affiliations:** ^1^Spinal Cord and Brain Injury Research Group, Stark Neurosciences Research Institute, Indiana University School of Medicine, Indianapolis, IN, United States; ^2^Department of Neurological Surgery, Indiana University School of Medicine, Indianapolis, IN, United States

**Keywords:** cell therapy, clinical trials, regeneration, myelination, functional recovery, biotherapeutic products, Schwann cells, spinal cord injury

## Abstract

The benefits of transplanting cultured Schwann cells (SCs) for the treatment of spinal cord injury (SCI) have been systematically investigated in experimental animals since the early 1990s. Importantly, human SC (hSC) transplantation for SCI has advanced to clinical testing and safety has been established via clinical trials conducted in the USA and abroad. However, multiple barriers must be overcome to enable accessible and effective treatments for SCI patients. This review presents available information on hSC transplantation for SCI with the intention to uncover gaps in our knowledge and discuss areas for future development. To this end, we introduce the historical progression of the work that supports existing and prospective clinical initiatives and explain the reasons for the choice of hSCs while also addressing their limitations as cell therapy products. A search of the relevant literature revealed that rat SCs have served as a preclinical model of reference since the onset of investigations, and that hSC transplants are relatively understudied, possibly due to the sophisticated resources and expertise needed for the traditional processing of hSC cultures from human nerves. In turn, we reason that additional experimentation and a reexamination of the available data are needed to understand the therapeutic value of hSC transplants taking into consideration that the manufacturing of the hSCs themselves may require further development for extended uses in basic research and clinical settings.

## Introduction

Over the past few decades, much progress has been made in the understanding of the pathology and molecular mechanisms of spinal cord injuries (SCI) and the design of assorted repair strategies in experimental animals. However, developing effective treatments for humans remains a challenge. Trauma to the spinal cord often results in the formation of fluid-filled cavities and the development of astrogliotic or fibrotic scar tissue that altogether create an inhibitory environment to axonal regeneration. For this reason, the addition of cells has been chosen as the main strategy to fill the cystic cavities, reduce scarring, and ultimately support axon regrowth. Schwann cells (SCs) are one of the most studied cell types for such purposes (reviewed in Tetzlaff et al., 2011; Bunge and Wood, 2012; Deng et al., 2015a). SCs are neuroglial cells that not only drive axon regeneration and myelination in the peripheral nervous system (PNS) but also perform an analogous function when transplanted into the spinal cord. In addition, SCs can be isolated from a patient’s own nerve and expanded *in vitro* prior to implantation, which makes them an outstanding cell type for autotransplantation therapy in SCI (Guest et al., 2013).

SCs have been transplanted experimentally for >40 years to exploit their proregenerative and myelinating functions in traumatic and demyelinating lesions in the PNS and central nervous system (CNS) (Blakemore, 1977; Duncan et al., 1981; Kohama et al., 2001). A long-standing history of work, including clinical investigations initiated over a decade ago, has established that SC grafting for spinal cord repair is translatable to humans. To date, studies involving the delivery of hSC transplants alone (Saberi et al., 2008, 2011; Zhou et al., 2012; Anderson et al., 2017; Gant et al., 2021) and together with other cell types (Chen et al., 2014; Oraee-Yazdani et al., 2016) have indicated no undesirable effects in human participants of clinical trials. Safety measures and cell dosage in patients with subacute and chronic SCI have been addressed via USA-Food and Drug Administration (FDA)-sanctioned trials (Anderson et al., 2017; Gant et al., 2021). Nevertheless, there are basic questions about the use of hSCs that have yet to be answered, as most supportive data for a role of SCs in SCI repair have been gathered using rodent cells mainly from rats (Tetzlaff et al., 2011). While rodent SCs have been regarded as useful in basic and preclinical studies, they may be insufficient to obtain conclusive information relevant to humans, as it is becoming increasingly clear that hSCs have special characteristics and only partially mimic the rodent counterparts (reviewed in Monje, 2020).

Therefore, the goal of this review is to evaluate current knowledge of hSC transplantation for SCI in an effort to identify prospects and challenges relevant to translational medicine. Research on rodent SC transplantation has been systematically reviewed and will not be described here in detail (for reference, see Oudega and Xu, 2006; Tetzlaff et al., 2011; Bunge and Wood, 2012; Deng et al., 2015a). In the sections below, we have explained the rationale for the choice of hSCs to repair CNS injuries starting with a description of the pioneering studies that supported the cell therapy strategy. We have addressed the advantages and limitations of hSC-based therapies, analyzed the available literature to uncover gaps in our knowledge, and discussed areas for future development or exploration. The ultimate goal of this review is to highlight some of the outstanding questions that need to be resolved to maximize treatments for human SCI, considering that the therapeutic approaches will likely evolve as new information is gained from basic and clinical work.

## Why Use Transplants of Cultured SCs for SCI?

Although SCs do not normally reside in the CNS, there is a strong rationale for using them in spinal cord repair because they provide a substrate for axonal growth and a means of replacing myelin (section “Therapeutic Action”). SCs are an abundant and accessible cell type amenable for culturing under Good Manufacturing Practices (GMP) and autologous introduction into the human body, thus alleviating the need for chronic immunosuppression (Bunge et al., 2017).

**FIGURE 1:**
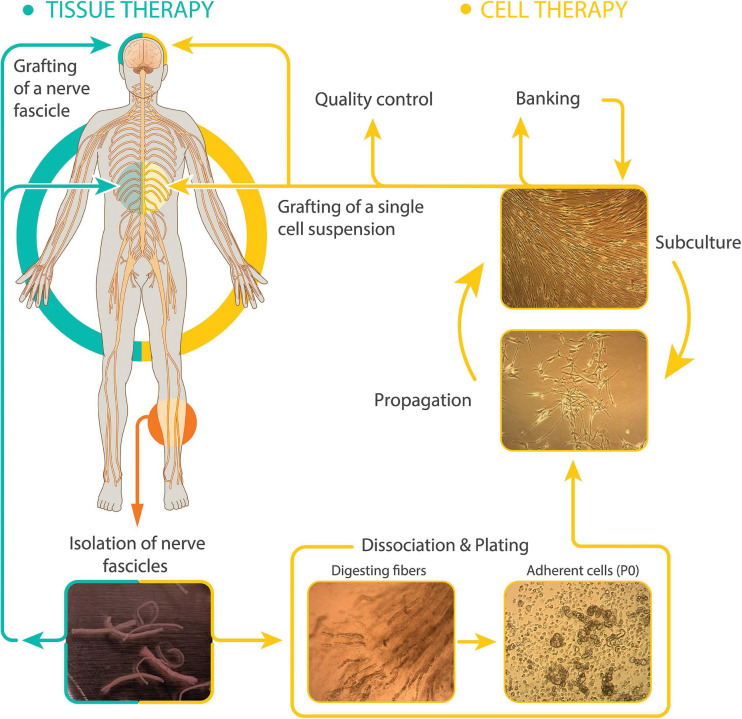
Modalities of hSC-based autotransplantation therapies for neuroprotection and regeneration in the CNS. Most clinical initiatives in SCI and other paradigms have used autologous cultured (right, cell therapy) or uncultured (left, tissue therapy) hSCs from sural nerve delivered within or around the injury area (diagram). The established hSCs are usually purified and expanded before collecting them as a single cell suspension for implantation. Delayed dissociation of pre-degenerated (cultured) nerve fibers induces SC activation and enhances recovery of cells at passage-zero (P0). Representative images of dissected sural nerve fascicles in culture medium (bottom left) and intermediate steps in the isolation and culturing of hSCs are shown. Culture is usually carried out up to passage-2 to generate hSCs ready to transplant and stocks for banking. Digested fibers: nerve fascicles during enzymatic dissociation. Adherent cells: primary cells right after isolation showing abundant myelin debris. Propagation (with mitogenic factors) and subculture: purified hSCs at low (lower image, subconfluent cells) and high density (upper image, confluent cells).

Therapeutically, SCs can be delivered in the following two essentially different modalities: (1) peripheral nerve grafts, i.e., undissociated cells within their connective tissue layers, and (2) purified cells in suspension obtained via *in vitro* culture techniques (Figure 1). Whereas uncultured tissue grafts have been chosen for nerve replacement in PNS lesions and neuroprotection in the brain (El Seblani et al., 2020), there is precedent for nerve autografting in the spinal cord of humans. Cultured SCs are preferred for the treatment of spinal cord trauma (Guest et al., 2013) and demyelinating diseases (Kohama et al., 2001) because isolated cells cause less damage during implantation and in turn, they give rise to a new and stable bridging tissue that usually adjusts well to the unique size and shape of spinal cord lesions (Guest et al., 2013).

The feasibility of implementing a hSC-based therapy using cultured cells was evidenced when methodological advancements made it possible to isolate and propagate hSCs from the nerve fascicles of adult donors to the high numbers needed for grafting. Once established *in vitro*, normal hSC populations conserve essential SC characteristics (Levi et al., 1995; Rutkowski et al., 1995; Casella et al., 1996; Weiss et al., 2016) and their capacity for undesirable differentiation or aberrant (cancer) growth is extremely low (section “Safety”). hSCs can be cryopreserved without loss of viability (Kohama et al., 2001; Bastidas et al., 2017) and genetically modified *ex-vivo* to enhance their potency or enable visualization post-transplantation (Haastert et al., 2007). While SC cultures from multiple sources have been examined in preclinical SCI research, the ones from mature (adult) nerves are preferred (sections “Safety” and “Types of Transplantable hSCs”), despite their limitations (section “Limitations of hSC Cultures”).

### Therapeutic Action

Mature SCs are endowed with remarkable plasticity. They rapidly convert into proliferative cells, often referred to as repair or activated SCs, in response to nerve (axonal) degeneration (Jessen and Mirsky, 2019). Repair SCs create an environment that is both neuroprotective and permissive to axon regeneration by virtue of the trophic factors, extracellular matrix (ECM) molecules, and adhesion molecules they express on their surface or release into the environment (Jessen and Arthur-Farraj, 2019). SCs established in cell culture retain functional capacity for nerve repair and (re)myelination. Once transplanted in the injured CNS, they can reduce the size of the lesions, attract and guide descending and ascending axons into the implant, and modestly improve locomotor function without additional interventions (reviewed in Fortun et al., 2009; Bunge and Wood, 2012; Deng et al., 2015a). Most importantly, transplanted SCs provide ensheathment and a myelin sheath to newly regenerated propriospinal axons as well as demyelinated axons resulting from secondary damage in spinal cord lesions (Deng et al., 2015b).

### Safety

SC-based transplants are typically safe. To date, most preclinical investigations on rodent and hSC transplantation have used cells from adult nerves, as it is anticipated that the individuals donating the tissue, and, in turn, receiving the cultured SCs, will mostly be adults (Tetzlaff et al., 2011; Bunge et al., 2017). Mature nerves comprise highly differentiated SCs in the form of ensheathing (Remak) and/or myelinating cells. It is worth mentioning that even though mature SCs dedifferentiate substantially after injury, they remain lineage-committed (unipotent) and cannot give rise to cells of an unrelated lineage (Jessen and Mirsky, 2019). SC precursors (SCPs) and neural crest cells are not evident in adult nerve tissues based on the data from rodents (Stierli et al., 2018). Although the cellular constitution of adult human nerves has not been investigated fully, it is unlikely that multipotent cells such as SCPs or stem cells are introduced in the culture workflow, which would be undesirable for transplantation due to the risk for uncontrolled growth or differentiation of the grafted cells.

Immortalization of SCs *in vitro* and lack of replicative senescence has been observed in cultures of rat SCs but not of human SCs with the consideration that immortalized rat SCs are often non-cancerous and able to maintain anchorage dependent growth (Mathon et al., 2001; Funk et al., 2007). Tumor formation by transplanted rat SCs has been reported for adult skin-derived (May et al., 2018) and postnatal nerve-derived SCs subjected to excessive passaging (Langford et al., 1988). Though these findings cannot be disregarded, the risk for acquired pluri- or multi-potency, transformation or unregulated growth of SCs pre- and post-transplantation are low regardless of whether the cells are derived from developing (embryonic, neonatal) or mature tissues. First, standard culture conditions are sufficient to allow undifferentiated SCPs to spontaneously give rise to SCs on schedule, resembling their differentiation process *in vivo* (Mirsky and Jessen, 2018). Second, hSC cultures are highly resistant to immortalize (Emery et al., 1999) and have a limited capacity to proliferate after being isolated from the nerves (Levi et al., 1995; Monje et al., 2018). No evidence of cancer development, excessive migration or mislocalization of the hSC transplants in contusive spinal cord lesions was found in preclinical investigations of donor nerve-derived hSCs established via standard methods (Bastidas et al., 2017). In sum, the stability of nerve-derived SC cultures, and the evidence indicating their safety for implantation, has greatly facilitated the clinical development of hSC-based cell and tissue therapies.

### Types of Transplantable hSCs

The sources of therapeutic hSCs can be varied because mature and developing tissues such as nerves, ganglia, or roots are suitable for the culturing of normal primary hSCs regardless of their anatomical location and donor-related characteristics (Crawford and Armati-Gulson, 1982; Rutkowski et al., 1992; Casella et al., 1996; Weiss et al., 2016). Preexistent trauma to the spinal cord does not preclude the derivation of quality-grade hSC cultures from the sural nerve, a sensory nerve most often used for autologous hSC isolation in clinical trials because of the ease of access and tolerability to the donors (Saberi et al., 2011; Aghayan et al., 2012; Anderson et al., 2017). Recent work has underscored that SCs lose their potency for nerve repair in aged animals (Painter et al., 2014). Nevertheless, empirical observations have not revealed major difficulties with the procurement and expansion of hSCs from the nerves of older adults. Optimized protocols that use mitogenic factors to achieve expansion of hSCs *in vitro* allow the preparation of proliferative hSC cultures from humans aged 60 years and above (discussed in Monje, 2020). Whereas donor age affects the intrinsic growth rate of hSCs in culture, age is not a restrictive factor for the preparation of healthy, expandable hSC cultures from cadaveric and organ donor tissues (Boyer et al., 1994; Bastidas et al., 2017). An important barrier to mass producing adult nerve-derived hSC cultures for transplantation is the recovery of enough numbers of viable, adherent hSCs at passage-zero (Figure 1). However, recovering expandable hSCs from pre-degenerated sural nerve fibers is not an issue of concern when autologous >10 cm long nerve explants are used together with standardized protocols for tissue dissociation, cell plating, purification, and expansion (Bunge et al., 2017). Indeed, the possibility to derive hSC cultures from the sural nerve of patients from a wide range of ages has facilitated clinical translation of hSC autotransplantation therapies in general (discussed in Guest et al., 2013; Bunge et al., 2017).

The skin is another potential donor tissue for hSC derivation. In addition to its richness in mature SCs associated with nerve terminals, the skin concomitantly possesses a reservoir of immature skin-derived precursors (SKPs) of neural crest origin capable of giving rise to SCs via directed differentiation (Biernaskie et al., 2007). Extensive preclinical data support the feasibility to obtain SC cultures from SKPs (rodents) and use them for spinal cord repair in subacute (Sparling et al., 2015; May et al., 2018) and chronic SCI models in rodents (Assinck et al., 2020). hSC cultures from skin biopsies exhibit strong neuroregenerative properties and a nearly identical transcriptional signature with respect to those derived from nerves (Stratton et al., 2017). However, the transplantation SKP-SCs can lead to uncontrolled proliferation and the formation of masses in the spinal cord based on data from adult rodent cells (May et al., 2018). SKP-hSCs have not been tested clinically, but the choice of skin rather than nerves for hSC procurement has an obvious advantage from an ethical standpoint.

Lastly, hSCs can be generated *in vitro* from embryonic and induced pluripotent stem cells via stepwise differentiation, or from somatic cells such as fibroblasts via transdifferentiation or direct conversion (reviewed in Huang et al., 2020). SC-like cells prepared from bone marrow- or adipose tissue-derived mesenchymal stem cells offer promise for autologous therapies to repair damage in SCI and other forms of nervous system trauma (Hopf et al., 2020). The technologies to generate SC-like cells from unconventional sources are advancing swiftly but uses in human subjects are controversial due to the instability, heterogeneity, and likely tumorigenic potential of the resulting cellular products (Lehmann and Hoke, 2016). SC-like cells of human origin may be valuable for preclinical research to gather patient-oriented data because they can be used as cellular models for genetic or pharmacological studies, toxicity screens, or other investigative approaches that can be accomplished without major ethical concerns or risks to the patients. A few noncancerous hSC lines have been created (Lehmann et al., 2012) but their use in transplantation and other functional studies is not generally recommended.

### Limitations of hSC Cultures

As summarized in Table 1 and discussed in other sections, the choice of hSCs from adult nerves also encompass limitations related to the properties of the cells themselves, the biomanufacturing methods used to prepare them, and the efficacy of the transplants (section “The Knowledge Gap in our Understanding of hSCs”). Purified SCs propagated *in vitro* are manipulated cells irrespective of their origin and mode of preparation. Consequently, multiple steps of quality control are needed to ensure the product is suitable for human use. For this reason, the hSCs intended to be used in participants of FDA-regulated clinical trials are scrutinized extensively to confirm their identity, purity, viability, stability and sterility prior to transplantation, as well as the absence of residual components from the manufacturing process (Anderson et al., 2017). In addition, the mode of action of hSCs in nerve repair is complex, and the resulting populations often vary from donor to donor and possibly also with passaging, which makes determination of potency (biological activity) quite challenging via *in vitro* and *in vivo* assessments.

**TABLE 1:** Advantages and limitations of cultured hSCs for grafting strategies.

	Advantages	Limitations
Origin and accessibility	hSCs are widely distributed and accessible cells in peripheral nerves and the skin.	A nerve segment needs to be sacrificed for cell isolation

Tissue procurement	Sources are varied. Tissues may be autologous or allogenic. Cadaveric biospecimens are also suitable.	Fairly invasive surgical intervention is needed for nerve harvesting with morbidity to the donor and risk of sensory deficits, pain and neuroma formation.

Cell processing	Feasible and fairly reproducible when optimized for GMP manufacturing.	Labor-intensive and time consuming (several weeks) to obtain enough cells with high associated costs. Isolation of primary cells may be compromised by low yields or poor viability.

Expandability	Significant amplification in culture is feasible. An excess of hSCs is often produced using nerve explants >10 cm in length.	Chemical mitogens and animal serum cannot be avoided. High lot-to-lot variability in total cell yields may be expected. Expansion over passage 4–5 is limited by senescence.

Purity	Achieving >98% hSC purity is feasible by standard methods.	Fibroblast contamination may be hard to control in certain populations. Clinically-relevant methods for effective cell purification need to be developed

Quality control	Fairly simple and straightforward phenotypic characterization by immunological methods.	Expanded populations are heterogeneous (donor-or lot-dependent). The quality/quantity of the populations can vary with subculture

Biological activity	Normal hSCs are proliferative, unipotent and phenotypically stable under established growth conditions.	Mechanism of action is complex. Biological activity is hard to determine and quantify by *in vitro* and *in vivo* assessments. Standard potency assays for hSCs are not available

Safety	No indication of transformation *in vitro* and *in vivo* (xenografts) has been reported.	Investigations are limited to a handful of studies. More data are needed.

Banking and transfer	Cryogenic storage can be implemented at any passage with high recovery after thawing.	Feasibility is contingent upon initial yields and expandability of the stocks.

*Most considerations provided in the Table are relevant to nerve-derived, established hSC cultures because of clinical relevance to transplantation in SCI and other conditions. Appropriate citations can be found in the text.*

## The Temporal Gap Between Basic and Clinical Science: From Rodents to Humans

The history of work on SC transplants is long-standing. By the early 1980s, it was clear that peripheral nerve tissues were supportive of long-distance axon regeneration from central neurons (Richardson et al., 1980). At that time, it was also feasible to recapitulate at least two of the constituents of the peripheral nerve that had an influence on axonal growth, i.e., the SCs and their associated ECM, in the simplified environment of a culture dish (Wood and Bunge, 1975). These early discoveries prompted an increased interest in the use of cultured SCs alone and together with ECM materials for the replacement of whole nerve grafts in animal models of trauma, neurodegeneration, and demyelination (Bunge, 1993).

The idea that cultured neuroglia—SCs in particular—could be used therapeutically to replace cells lost due to nervous system injury or disease was framed in a 1975 review article that highlighted progress on neural culture systems at that time (Bunge, 1975). This concept was supported by *in vitro* observations showing that SCs survived well without neurons and that their proliferation was elicited by axon membrane-bound molecules (Wood and Bunge, 1975). The discovery of defined soluble mitogenic factors for use as axon-mimetics in the ensuing years (Raff et al., 1978) confirmed that SCs obtained directly from nerve tissues remained proliferative over several passages in the absence of other cell types (Brockes et al., 1979). Since then, additional innovations allowed for the preparation of SC cultures at rising scales from mature and developing tissues as well as from different species, including humans, thereby providing researchers with an opportunity to begin exploratory research on SC grafting in the 1980s.

The goal of the earliest cultured SC transplants was to restore myelin rather than repair tissue affected by trauma in the CNS. By transferring cultured rat SCs together with their collagen substrate into demyelinating lesions in the spinal cord of immunocompromised mice (Duncan et al., 1981) and myelin-deficient rats (Duncan et al., 1988), researchers learned that SCs established in cell culture retained differentiation potential *in vivo*. Duncan et al. (1981) provided the first evidence on the engraftment of cultured SCs in a xenogeneic host and their association to myelin sheaths that exhibited features of peripheral myelin (Duncan et al., 1981). Regarding SCI repair, researchers used complex 3-dimensional scaffolds containing embryonic SCs and neurons from sensory ganglia (Kuhlengel et al., 1990b) prior to applying purified SCs in suspension from the nerves of adult rats (Xu et al., 1995) and humans (Guest et al., 1997b) to bridge sites of injury in the spinal cord (sections “Development and Testing of Non-human SC Grafts” and “Development and Testing of hSC Grafts in Immunodeficient Rodents”). These ground-breaking investigations sparked a wave of research in the following decade to test the efficacy of SC transplants in various animal models of SCI, understand the underlying mechanisms of repair, and find ways to improve functional outcomes via combinatorial treatments (Fortun et al., 2009; Bunge and Wood, 2012; Deng et al., 2015b). Nevertheless, it should be noted that transplantation of hSC products in SCI patients was not attempted until recently, with the first clinical study reported in year 2008 (Saberi et al., 2008) (section “hSC Transplantation in Human Subjects”).

**FIGURE 2:**
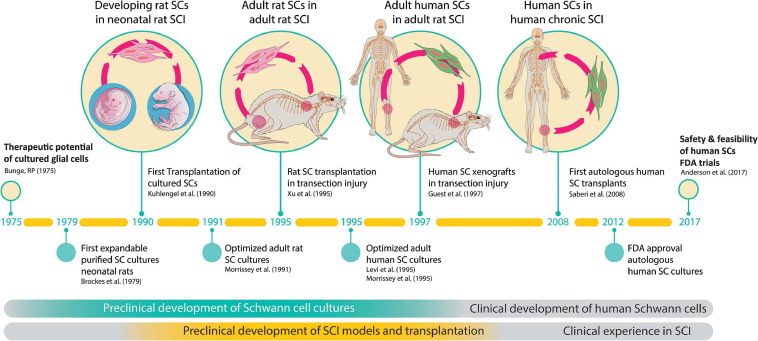
The path to clinical translation of SC transplantation in SCI. The timeline summarizes milestones and selected major achievements in support of the overall strategy. The history of work explaining progress made since the conception of the original idea (Bunge, 1975) to the completion of the first FDA-sanctioned trial is described in the text. Much work was done in the initial phases to (i) develop technologies for SC culturing, and (ii) obtain proof-of-concept data of SC transplants in animal models of SCI. Notice the 20-year gap between the first reported animal study (Kuhlengel et al., 1990b) and the first clinical study (Saberi et al., 2008). FDA approval for the first safety trial in the USA was gained in 2012 (Xu, 2012) and the results released in 2017 (Anderson et al., 2017).

Altogether, the work supportive of SC-based therapies has spanned nearly 5 decades and the transplantation strategy has been validated independently using SCs of various origins. Clinical trials have been initiated but this work is still in progress (Bunge et al., 2017). Key accomplishments pertaining to the preclinical development and testing of SC cultures in animal models of SCI and the onset of clinical investigations are summarized in Figure 2.

### Development and Testing of Non-human SC Grafts

Compelling data on SC transplants were presented in 2 companion publications encompassing the development of SC-containing cellularized scaffolds (Kuhlengel et al., 1990a) and their implantation into thoracolumbar lesions created in the spinal cord of newborn rats (Kuhlengel et al., 1990b). To obtain these scaffolds, cultures of dissociated embryonic dorsal root ganglion (DRG) neurons established on a collagen substrate were deprived of non-neuronal cells prior to being seeded with purified SCs from embryonic DRGs for the re-establishment of SC-neuron interactions and repopulation of the neuritic network. These co-cultures were loosely detached from their dishes and rolled up to create a scroll-like structure that was sectioned into small segments for further culture in the absence or presence of leptomeningeal cells to allow *in vitro* maturation of axon-associated SCs with and without vascular components. The reconstituted SC-containing scaffolds were in turn placed into cavities created by the removal of dorsal columns immediately after or at a delayed time point with respect to the injury event. The results indicated that the SC transplants filled the vacated spinal cord tissue, that both neurons and SCs survived for several months, and that the SC-grafts became well-vascularized *in vivo* without the contribution of leptomeningeal cells. Importantly, less differentiated grafts consisting of non-myelinating SCs without ECM components, and delayed implantation with respect to the injury event, allowed for a better fusion of the implants with the spinal cord.

Advancements in the production of large numbers of pure rat SCs from the mature sciatic nerve (Morrissey et al., 1991) allowed researchers to obtain unequivocal evidence of axon regrowth and myelination in allogenic or autologous SC-containing implants. A study by Xiao-Ming Xu et al. (1995) reporting the grafting of SC-seeded guidance channels into the transected cord of adult inbred rats was the first to show that cultured adult purified SCs suspended in a Matrigel matrix contributed to the formation of a vascularized cable tissue that supported the growth of adjacent propriospinal and sensory axons. This study further indicated that SCs alone could exert an axon growth-promoting function supportive of SC differentiation (myelination) in the spinal cord bridge without the physical and trophic support provided by transplanted sensory neurons though it was noticed that most of the SCs in the graft were associated with unmyelinated fibers (Xu et al., 1995).

### Development and Testing of hSC Grafts in Immunodeficient Rodents

By the early 1990s, researchers could generate mitotically active hSC populations using pediatric and adult nerves (Rutkowski et al., 1992) as the lifespan of cultured hSCs could be extended significantly with the addition of recombinant glial growth factor (GGF or neuregulin) and cAMP-stimulating agents such as cholera toxin and forskolin (Levi et al., 1995; Rutkowski et al., 1995). Suspensions of hSCs prepared from pre-degenerated nerves and expanded *in vitro* with the aid of the aforementioned chemical factors were grafted into the transected sciatic nerve (Levi et al., 1994b) and the spinal cord (Guest et al., 1997b) of nude (athymic) rats, a xenograft-tolerant strain, to assess their potential for nerve repair and myelination in the PNS and CNS, respectively. Guest et al. (1997b) used semipermeable guidance channels to create solid grafts containing purified hSCs embedded in Matrigel. When these grafts were implanted across a gap in the spinal cord at the thoracic level, interdigitated interfaces resembling CNS/PNS transition zones formed between the hSC grafts and the host spinal cord. Furthermore, supportive evidence was obtained that propriospinal and DRG axons extended projections into the implants (Guest et al., 1997b), as shown for rat SCs (Xu et al., 1995).

### hSC Transplantation in Human Subjects

Saberi et al. (2008, 2011) reported the first study on autologous hSCs transplantation in 33 patients with chronic SCI who had sustained thoracic and cervical injuries. These patients received several injections of a hSC suspension (sural nerve) within and on the sides of the lesion. The hSCs were prepared in the absence of mitogenic factors and animal serum, as autologous serum was used in replacement of the fetal bovine serum (Aghayan et al., 2012) that is a regular component of the hSC culture medium (Casella et al., 1996). No cases of permanent neurological worsening, increment in cavity size, abnormal tissue or tumors (as per contrast-enhanced MR imaging), or infectious complications were found in the 2 years that followed treatment. An independent study of 6 participants who received injections of autologous, activated SCs (purified, in suspension) on each side of the lesion reported no changes of concern as determined during the 5-year follow up period. Some signs of improvement in autonomic, motor, and sensory function in these patients were also recorded (Zhou et al., 2012). In this study, the hSCs were isolated from sural nerves that were ligated 1 week prior to tissue harvesting with the expectation that this procedure would increase the repair features of the isolated hSCs (activation) once established in culture.

In two additional trials, hSCs were given in a co-transplantation approach with other cell types, again confirming that the strategy is safe. In one study, patients with chronic SCI received adult hSCs (autologous, sural nerve) together with bone marrow mesenchymal stem cells through cerebrospinal fluid (Oraee-Yazdani et al., 2016). In the other study, embryonic SCs (heterologous, sciatic nerve) were transplanted together with olfactory ensheathing cells (Chen et al., 2014).

A team of scientists at University of Miami conducted two Phase I, FDA-regulated trials on hSC (sural nerve, expanded) autotransplantation for subacute (Anderson et al., 2017) and chronic SCI (Gant et al., 2021). The former was an unblinded, open-label, non-randomized, non-placebo controlled, dose escalation trial to test the safety and feasibility of obtaining and delivering autologous hSCs into the injury epicenter in 6 participants. One-year post-implantation, no serious neurological complications were associated with the transplanted cells, the spinal surgery or the cell injection system used (Anderson et al., 2017) which was designed by the same team to gently deliver cells to the spinal cord (Santamaria et al., 2018). The second trial was designed to use an individualized cavity-filling dose of hSCs in 8 patients with chronic cervical and thoracic injuries receiving rehabilitation before and after cell implantation (Gant et al., 2021). Not only did this trial confirm the safety of the approach but it also provided evidence of improvement in motor and sensory function in one individual.

## The Knowledge Gap in Our Understanding of HSCs

**FIGURE 3:**
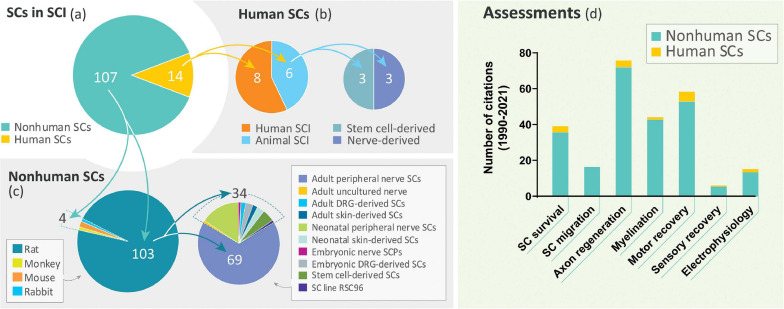
Literature review on SC transplantation in SCI. A PubMed search using the terms “spinal cord injury” and “Schwann cell transplantation” as entry key words retrieved only 14 publications on the transplantation of hSCs (diverse origin) in non-clinical and clinical settings during the period 1990–2021 **(a,b)**. This time frame was selected to take into consideration that the first published study on SC transplants in SCI dates from 1990 (Kuhlengel et al., 1990b). Our search did not discriminate whether the SCs were nerve- or stem cell-derived but we excluded studies on *ex-vivo* transplants. Studies on non-human SCs from a variety of sources and developmental stages were extensive (88.4%) but the use of adult tissue-derived rat SCs was prevalent **(c)**. A categorization of available studies according to the type of assessments performed in basic and clinical studies combined is shown in **(d)**. The total number of scrutinized studies in the most relevant categories are noted in the pie charts.

Despite the long history of work on SC transplantation for SCI (section “The Temporal Gap Between Basic and Clinical Science: From Rodents to Humans”), studies on hSCs are surprisingly scarce. A literature search covering the past 30 years revealed a dramatic disparity in the number of indexed publications reporting research conducted using hSCs with respect to non-human SCs in SCI models of transplantation (Figure 3a). A prominent observation was the widespread use of rat SCs mostly from adult nerves in preclinical research (Figure 3c), as noted previously (Tetzlaff et al., 2011). While most hSC studies consisted of clinical investigations, fragmentary literature exists regarding hSC xenotransplants (Figure 3b). We identified only 6 studies reporting results from nerve- and stem cell-derived hSC transplantation in adult nude rats (Guest et al., 1997a, 2005; Bastidas et al., 2017), Sprague Dawley rats (Babaloo et al., 2019), and Wistar rats (Kamada et al., 2005) using dorsal hemisection (Babaloo et al., 2019), transection (Guest et al., 1997a, 2005; Yan-Wu et al., 2011) and contusion injuries (Kamada et al., 2005; Bastidas et al., 2017). There were no reports of hSC transplantation in large animal models of SCI. A handful of publications on the xenografting of hSCs (nerve-derived, primary or expanded) in peripheral nerves (Levi and Bunge, 1994; Levi et al., 1994b; Emery et al., 1999; Hood et al., 2009; Stratton et al., 2017) and demyelinating lesions in the CNS (Brierley et al., 2001; Kohama et al., 2001) are informative on basic properties of the hSCs such as survivability, tumorigenicity and scarring but the available data on particular outcome measures for SCI are rather incomplete (Figure 3d). For instance, whether hSCs proliferate and differentiate in the injured spinal cord remains relatively unexplored. Factors contributing to engraftment success and functional improvement are unknown for hSCs. What seems evident from the examination of the available data (Table 2) is that critical discrepancies exist in the expected responses of rat SCs and hSCs both in anatomical (section “Anatomical Changes”) and behavioral (section “Functional Recovery”) measures.

**TABLE 2:** Comparison of the rat and hSC responses in spinal cord lesions.

Assessments	Results	
Survival and proliferation	Rat	A proportion of the grafted cells proliferate and survive in the long-term though there are discrepancies in independent studies regarding the extent of survivability in the initial stages
	Human	Variable rate of hSC survival (lot-to-lot) but poor overall in contusion injuries with a low proliferation rate (Bastidas et al., 2017)

Migration and localization of the transplants	Rat and human	Cells are restricted to the site of implantation. SC migration around the lesion can be improved with treatments in rat SCs but no information is available for hSCs

Axon growth into SC implants	Rat	Robust ingrowth of sensory and propriospinal axons into the SC graft. Brainstem axon growth has been reported in contusion and transection injuries but axons remain confined to the injury site unless the transplants are supplemented with additional factors
	Human	Axon growth is contingent upon graft survival (Bastidas et al., 2017). Sensory and propiospinal axon regeneration into hSC grafts has been reported. Motoneuron and long-track (brainstem) regeneration were observed in the transection model (Guest et al., 1997b)

Glial scar	Rat	Well-developed in the interface between the spared CNS tissue and the SC graft with a clear boundary between astrocytes and the SC graft unless treatments are provided to reduce environmental inhibition
	Human	Relatively good integration of hSC graft -spinal cord in contusion and transection injuries (Guest et al., 1997b). Glial scar is not evident around the hSC transplant. Intermingling hSC/astrocytes were seen in a few transplants in the contusion model (Bastidas et al., 2017)

Endogenous SC response	Rat and human	Abundant host-derived SCs develop in the lesion site and intermingle with the grafted cells. Studies on hSCs have not quantitatively addressed the contribution of endogenous SCs in the grafts

Ensheathment and Myelination	Rat	Abundant myelin of PNS origin is found in the SC-grafts. Grafted and endogenous SCs provide ensheathment to axons and form myelin within the grafts though variation is expected in independent studies
	Human	Myelin develops in hSC grafts but most fibers are non-myelinated (Guest et al., 1997b). PNS-like myelin in hSC grafts is likely host SC-derived (Bastidas et al., 2017)
Stability of the transplants	Rat	Long lasting (>6 months) and apparently stable unless signs of immune rejection are evident
	Human	Variable and batch-dependent (Bastidas et al., 2017) or not investigated

Tumorigenicity	Rat	Reported for skin-derived SCs but generally not observed using nerve-derived SCs
	Human	Not observed in CNS and PNS models of xenografting (Emery et al., 1999; Bastidas et al., 2017)

Functional outcome	Rat	Modest but significant motor and sensory improvement has been reported (various tests) though variable outcomes are possible in the absence of additional therapeutic interventions
	Human	Some improvement (in transection injury) or not determined (in contusive SCI), (Guest et al., 1997b)

Therapeutic enhancement	Rat	SC-elicited responses such as survivability post-transplantation, axon growth, and functional recovery can be improved with appropriate combination treatments
	Human	Fairly unexplored with the exception of increased astrocyte migration into SC grafts with daily infusions of IN-1 antibody and reduction of CST die-back with delivery of acidic FGF/fibrin glue (Guest et al., 1997a,b)

*A general comparison is made based on existing literature on hSCs, as cited in the text. It should be noted that up to date, there are no published studies in which hSCs and rat SCs, or SCs from any other species, were compared side-by-side. References to rat SCs are not listed in the table due to the vast number of studies that have focused on the subject matter (Oudega and Xu, 2006; Lavdas et al., 2008; Fortun et al., 2009; Tetzlaff et al., 2011; Bunge and Wood, 2012; Deng et al., 2015a; Bunge et al., 2017).*

### Anatomical Changes

Bastidas et al. (2017) reported the first hSC transplantation study in contusive SCI (Bastidas et al., 2017), a model that mimics clinical SCI, in support of an FDA-approved clinical trial led by the same team (Anderson et al., 2017). This study encompassed a comprehensive investigation of hSC grafts in the spinal cord of nude rats on the basis of comparing independent batches of GMP-compliant, mitogen (neuregulin/forskolin)-expanded hSCs from fresh isolates and cryogenic stocks. By showing that clinical-grade hSCs (with or without cryopreservation) remained confined to the site of implantation, did not form tumors, and promoted axon ingrowth into the contusive lesion, the researchers confirmed that the cells for use in patients were bioactive and safe. However, the authors noted that hSCs proliferated slowly and the grafts contained less than 10% of the total transplanted cells. In addition, the transplanted hSCs seemed not to directly contribute myelin in the grafts and there was a high degree of lot (or donor) variability in the rate of survival and other measures of anatomical repair including CNS tissue sparing, cavitation, and axonal extension. While hSC transplants contained abundant myelinated fibers of PNS origin, the myelin sheaths were likely host-derived, as immunoreactivity for human myelin-specific antigens within the engrafted hSC populations could not be confirmed (Bastidas et al., 2017).

To conclude, the low number of studies in which hSCs have been investigated so far, and the high variability displayed by individual hSC batches, has made it difficult to ascertain the properties of hSCs *in vivo*, especially pertaining restorative changes in the spinal cord. Evidence for repair and (re)myelination mediated by endogenous SCs that either migrate or differentiate locally in the lesion area where hSCs are transplanted has been shown consistently (Guest et al., 1997a; Bastidas et al., 2017), aligned with reports on rat SC transplants from nerve (Hill et al., 2006) and skin (Assinck et al., 2020).

### Functional Recovery

Although the data are varied and not always consistent regarding the extent of functional recovery elicited by transplanted rat SCs alone, partial recovery of motor and sensory function has been recorded when SCs are provided with other therapeutic treatments (Bunge and Wood, 2012; Deng et al., 2015a). Surprisingly, we found no published literature on the functional effect of nerve-derived hSCs (primary or expanded) in experimental animal models of SCI with the exception of one study by Guest et al. (1997b). Modestly higher scores during open field and incline plane testing in transected spinal cords were reported in animals that received bridging hSC-enriched grafts (Guest et al., 1997b).

## Challenges and Open Questions

As explained above, substantial progress has been made in translating SC therapies from bench to bedside. Experimentally, SCs have been transplanted alone and together with other cell types, in suspension and as part of complex (pre-assembled) cellular systems, with or without biomaterials, and additional experimental treatments, with general positive outcomes. As discussed in section “Understanding the Therapeutic Action of hSCs,” understanding how hSCs promote repair is critical to improve current and prospective approaches to the treatment of SCI, as it is widely recognized that optimization of hSC-based therapies will require more than one bioactive component. Multiple adjunct therapies can be applied to boost the neural supportive features of non-human SCs, to increase their migration capacity and integration with endogenous spinal cord cells, and/or to change the lesion microenvironment in order to reduce the glial scar around SC transplants to make it more permissive for axon growth (reviewed in Fortun et al., 2009; Bunge and Wood, 2012; Deng et al., 2015a). However, whether adjunct therapies would be effective with hSCs cannot be inferred from the available data, as no direct comparison exists on the relative potency of hSCs with respect to those of other species.

Likewise, some limitations of the strategy must be considered. Producing clinical-grade hSCs not only requires a sophisticated infrastructure but also a great deal of time and labor to obtain a transplantable batch. Multiple steps of quality assurance are needed to ensure the product itself is safe, as chemical mitogens, animal serum, recombinant laminin, proteolytic enzymes, and other factors are introduced in the culture workflow. Perils that are communicated to patients during the informed consent process include the possibility that an ineffective isolation of the cells, or the cultures being of insufficient quality or quantity, may preclude delivery of treatment (Guest et al., 2013). These and other potential obstacles are discussed in section “Expanding Access to Transplantable hSC Products.” In this section, we reason that future refinements of hSC-based therapies should involve a reduction of the operating time, the risks of the procedure, and the costs per-batch to enable more widespread patient access to these therapeutic products.

### Understanding the Therapeutic Action of hSCs

The myelin-forming capability of SCs has been essential to the formulation of a therapeutic hypothesis supportive of progression to clinical trials for SCI (Guest et al., 2013). However, functional myelination by engrafted hSCs in CNS lesions remains to be confirmed, as immunological data have shown that culture-expanded hSCs form myelin in the damaged peripheral nerve (Levi et al., 1994b) rather than in the spinal cord (Bastidas et al., 2017). Demonstrating myelination by hSCs in co-culture with DRG neurons (reconstituted systems) has proven problematic but *in vitro* models are insufficient to ascertain whether hSCs established in culture are inherently deficient to myelinate axons or whether the environment provided by the culture dish is insufficient to support their effective differentiation into myelinating cells (Morrissey et al., 1995; Monje et al., 2018). Multiple cell-extrinsic and cell-intrinsic factors can affect hSC (re)differentiation within CNS lesions. Competition with host SCs for access to an appropriate axonal substrate may limit the myelination efficiency of engrafted hSCs. Endogenous SCs colonize and even myelinate axons around chronic traumatic lesions in the spinal cord of humans (Guest et al., 2005) and this response may be enhanced by the transplantation of SCs, as evidenced in the rodent data (Hill et al., 2006). In addition, the isolation and expansion of hSCs may alter the (re)differentiation capability of the cells, as subculture moderately reduces myelination by transplanted hSCs in the PNS of immunodeficient rodents (Levi and Bunge, 1994; Levi et al., 1994b).

Whereas hSC grafts do not evoke undesirable effects in humans or experimental animals, it is unclear whether consistent engraftment and long-term survival of hSCs can be achieved experimentally. Therapies to prevent cell loss after transplantation may be needed. hSC survival in contusive injury (nude rats) was reported to be variable and low (Bastidas et al., 2017). This finding is aligned with reports that revealed that a majority of transplanted rat SCs die acutely post-transplantation, mainly due to necrosis and to some extent also due to apoptosis (Hill et al., 2007); however, grafts of rat SCs remain stable for >7 months and proliferation may occur as early as 1-day postgrafting (Wang and Xu, 2014). One caveat is that hSCs are antigen-presenting cells and may be sufficient to evoke an immunogenic response. hSC cultures established *in vitro* upregulate the expression of the major histocompatibility complex II (MHCII) (Weiss et al., 2016), which together with MHCI has been linked to the rejection of peripheral nerve allografts (Ansselin and Pollard, 1990).

Each population of transplantable hSCs is unique and a high degree of batch variability is expected, possibly due to, but not simply because of, patient-specific characteristics. Attributes that are specific to each donor are not usually evident when the cells are established in culture, but their impact may be substantial with longer survival times (Monje et al., 2018). Assorted factors can influence the bioactivity of individual cell batches, and undesirable effects on host tissues such as excessive astrogliosis or inflammation may occur. For instance, the presence of nerve-derived fibroblasts in hSC cultures has resulted in the deposition of large amounts of collagen and extensive axonal degeneration when transplanted in areas of acute demyelination in the spinal cord of adult rats (Brierley et al., 2001). Whether human fibroblasts are seemingly deleterious in traumatic SCI is unknown, but we suspect the fibroblast populations to be supportive of nerve repair because of their immaturity and their strong proregenerative transcriptional signature (Peng et al., 2020). It should be mentioned that SCs alone are usually ineffective to favorably modify the glial scar. However, evidence has pointed out that hSCs intermingle with astrocytic process from a xenogeneic host and create more permissive host-graft interfaces in the spinal cord when compared to rat SCs (Bastidas et al., 2017).

To conclude, it is expected that hSCs are beneficial irrespective of initial survival rates and donor variability but the functional consequences of hSC transplantation are ill-defined. Discriminating between true axon regeneration, i.e., axonal growth into the cavity filled with grafted hSCs, and sparing in contusive SCI can help in interpreting functional outcome. How to improve the therapeutic value of hSCs prior to and after transplantation remains an area for exploration and development.

### Expanding Access to Transplantable hSC Products

For decades, basic and translational research on hSCs has been hampered by the overall lack of technologies to access and understand the cell material itself. First, the methods for processing animal nerve-derived cells are ineffective for humans, and second, the properties of cultured hSCs differ from those of other species (Monje, 2020). Preparing and maintaining hSC cultures over several weeks require sophisticated knowledge and a tailored management of culture variables to achieve sufficient cell yields at a high level of purity. Additionally, hSCs have a limited lifespan in culture and need to be prepared each time they are required unless methods are optimized to produce large enough stocks for cryogenic storage.

Even with protocols and expertise in place, one should not underestimate the impact of the high costs associated with hSC bioprocessing due to the expensive materials (including GLP-grade chemicals), dedicated personnel, and quality assurance for each individual preparation. Though autologous and freshly prepared products are the gold standard for patients, cryopreserved hSCs may be considered to increase hSC availability in preclinical research (Bastidas et al., 2017). It should be noted that hSCs can be prepared from cadaveric nerves (Boyer et al., 1994) and tissues that would otherwise be discarded during surgical procedures, such as plastic or reconstructive surgeries, amputations (Kohama et al., 2001), and aborted embryos. There is one precedent for the use of fetal (sciatic nerve) hSCs for the treatment of chronic SCI in humans (Chen et al., 2014). Using established practices, like safe cold storage of tissue biospecimens prior to cell processing (Levi et al., 1994a), can certainly aid in getting access to more varied or even non-local sources of human tissues for hSC isolation. The banking of hSCs may be considered in cases where an excess of cells is generated, thereby allowing the transfer of the cells elsewhere, or delayed use for reanalysis or redosing. Regrettably, SC-like cells from stem cells or other sources are not suitable for near-term clinical cell therapy. Important hurdles need to be overcome from the biological and regulatory standpoints for SC-like cells to be considered appropriate for human transplantation.

Limits to the expandability of hSC cultures may vary but assorted strategies can be implemented to expedite cell processing, enhance the recovery of cells, and optimize (or standardize) the quality of the preparations. With the development of pertinent innovations, such as microcarrier-based bioreactor systems, individual hSC cultures can be scaled-up significantly (Mirfeizi et al., 2017). Expedited processing of adult rat and human SC cultures can be achieved using protocols involving the immediate dissociation of nerve fibers (Andersen et al., 2016; Weiss et al., 2016). The removal of animal serum and mitogenic factors from the culture medium has already been implemented for clinical grade cultivation of hSCs (Aghayan et al., 2012). Indeed, serum-free processing (Mirfeizi et al., 2017) and other modifications of the standard protocols can ease regulatory restrictions for quality control and product release.

Alternatively, peripheral nerve tissues (uncultured) obtained as same-day products without (or with minimal) manipulation can be prepared expeditiously and used with negligible risk to patients. Autologous nerve grafts have been used to bridge a gap in the spinal cord in combination with autologous olfactory ensheathing cells and fibroblasts (Tabakow et al., 2014), and bone marrow-derived mesenchymal stem cells (Amr et al., 2014) in human subjects. Over 70 patients received implants of autologous unprocessed sural nerve fascicles in the brain as part of a combinatorial treatment for Parkinson’s disease with no reported complications (El Seblani et al., 2020). Though the implantation of unprocessed nerves is usually discouraged for SCI, no evidence indicates that it may be unsafe in humans. An advantage of autologous tissues over cultured cells is that the former products are exempt from regulatory constraints and can offer a safer and cost-effective alternative for implantation of hSCs in the CNS (El Seblani et al., 2020). Finally, whole cells may not be needed, as diverse subcellular products such as SC-derived exosomes may have sufficient therapeutic effects in promoting axon growth (Lopez-Leal et al., 2020).

## Concluding Remarks

The expectations of the scientific community and the general public regarding the outcome of cell therapy trials are high in view of the lack of available options to treat human SCI and other types of CNS damage. Human studies have shown that transplanted hSCs are well tolerated in the spinal cord. Neurological improvement has been insinuated in some patients but clinical trials have not advanced to efficacy testing. As described above, investigations on hSC transplants are lagging, and crucial questions on the properties of hSCs *in vivo* remain unanswered. On the one hand, additional systematic experimentation and replication studies using hSCs are warranted to confirm (or disprove) the idea that rodent SC transplants are useful models for the development of hSC-based therapeutics. On the other hand, the established hSC transplantation paradigm using cultured cells obtained via traditional methods may pose a barrier to a widespread use of hSCs in basic science and in the clinic. Current technologies for hSC manufacturing restrict operations to centers equipped with considerable financial and scientific capabilities and this may affect the translation of the strategy in the longer term, especially for extending the treatment to large patient cohorts. Clinical initiatives involving cell therapies for SCI are at risk to be halted at an early stage due to the financial burden (Scott and Magnus, 2014). Simplification of the current transplantation strategy is possible because there are alternative ways to take advantage of the extraordinary regenerative capacity of peripheral nerves, in particular that of hSCs, for the development of novel biotherapeutics. Targeted innovations involving banked cells, uncultured or minimally manipulated nerve tissues or cells, and even extracellular vesicles may facilitate the development of additional products, including off-the-shelf ones, for preclinical, and perhaps clinical, applications.

To conclude, the history of work on SC transplants has highlighted the multiplicity of approaches attempted experimentally to exploit their value for SCI repair. We believe it is pertinent and timely to build upon existing knowledge, and the promising clinical results, to revitalize research on hSC therapies. Future directions toward this goal may encompass the creation of alternative hSC-based products for use alone or in support of other therapeutic approaches.

## Author Contributions

PM: conceptualization, writing, figure preparation, data analysis, and funding. LD: data collection and analysis, and manuscript editing. X-MX: manuscript editing and funding. All authors contributed to the article and approved the submitted version.

## Conflict of Interest

PM is founder of GliaBio LLC, a consulting company that focuses on glial cells. The remaining authors declare that the research was conducted in the absence of any commercial or financial relationships that could be construed as a potential conflict of interest.
